# Molar Uprighting: A Considerable and Safe Decision to Avoid Prosthetic Treatment

**DOI:** 10.2174/1874210601711010466

**Published:** 2017-08-31

**Authors:** Taísa Boamorte Raveli, Dirceu Barnabé Raveli, Kelei Cristina de Mathias Almeida, Ary dos Santos Pinto

**Affiliations:** 1 Orthodontics Department, Faculty of Dentistry, São Paulo State University, Avenida Portugal, 887 – CEP 14.801-075 Araraquara-SP Brazil; 2 Orthodontics Department, Faculty of Dentistry, São Paulo State University, Rua Humaitá, 1680 – CEP 14.801-903 Araraquara-SP Brazil

**Keywords:** Molar uprighting, Tooth movement, Adult. tooth loss, Orthodontic appliances, Biomechanics

## Abstract

**Background:**

Tipped lower molar over edentulous space is very common in orthodontics practice when adults seek treatment. The segmented arch technique features a predictable force system that provides a controlled release of force that can produce light and continuous tooth movement.

**Case Description:**

A female adult patient, who lost a permanent lower first molar, needed correction of the position of her permanent first molar place. Instead of making space for rehabilitation, it was closed after second molar uprighting and a balanced interdigitation was created without prosthetics. The patient was successfully treated with segmented arch technique using root correction spring activated with geometry VI to promote uprighting of a tipped molar and Niti spring coil to promote space closure.

**Practical Implications:**

Segmented arch technique is known to provide predictable light and continuous forces, which is very much indicated in adult treatment. There are several things to consider when orthodontically treating adult patients. Their periodontal conditions might not be ideal, less bone apposition may occur, and side effects of orthodontic tooth movement are expected. Thus, a predictable and controlled orthodontic treatment is needed.

## 1 INTRODUCTION

Adult patients with early loss of lower first permanent molar are frequent in orthodontics practice. The consequence for this early loss is the second permanent molar to tip mesially into the space of the lost tooth [1-3]. Other effects of early loss of a mandibular first molar are premolars distally drifted, extrusion of upper molars, constriction of the edentulous ridge causing altered gingival form, infrabony defect mesial to the inclined molar, stepped marginal ridges, food impaction and posterior bite collapse [3-5]. Pseudo-pockets form adjacent to the tipped tooth since bone contour follows the cementoenamel junction [6] and in adults, the major concern in moving the tipped tooth into the right position is to maintain periodontal tissues and avoid extrusion when applying an orthodontic force [7-9].

There are some ways to place the tipped molar into its correct position: use of cantilevers, known to provide best controlled movement [7]; straightwire associated with spring coil, a statically indeterminate system that cannot be quantitatively defined, [1] and more recently the use of miniscrews associated with straightwire.

**Fig. (1):**
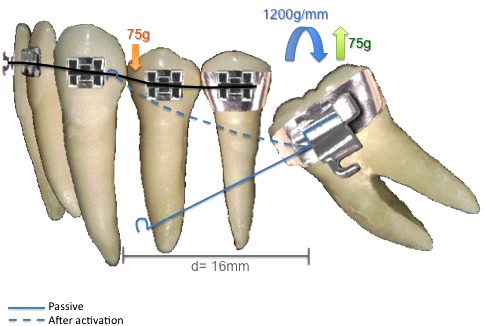
One cantilever force system where the loose end of the cantilever is attached to the stainless steel wire between canine and first bicuspid. This loose end when attached promotes a downward force with 75g of magnitude; while the inserted end will promote an upward force of equal magnitude, promoting molar extrusion as a side effect.

**Fig. (2):**
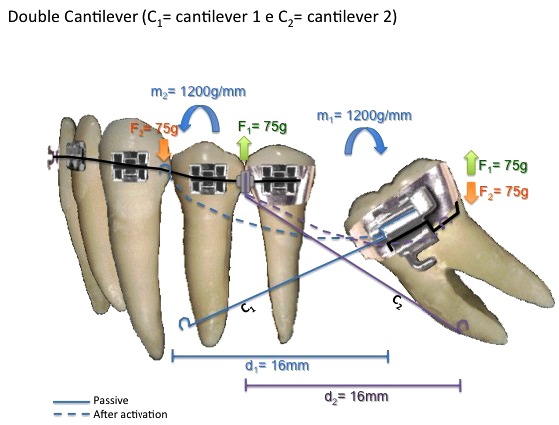
Two cantilevers force system has the first cantilever working as the single cantilever force system, and because there is an extrusion side effect, a second cantilever is placed with the inserted end between bicuspids and the loose end attached to a piece of stainless steel wire at the band. This way, the second one cancels the extrusion caused by the first cantilever.

**Fig. (3):**
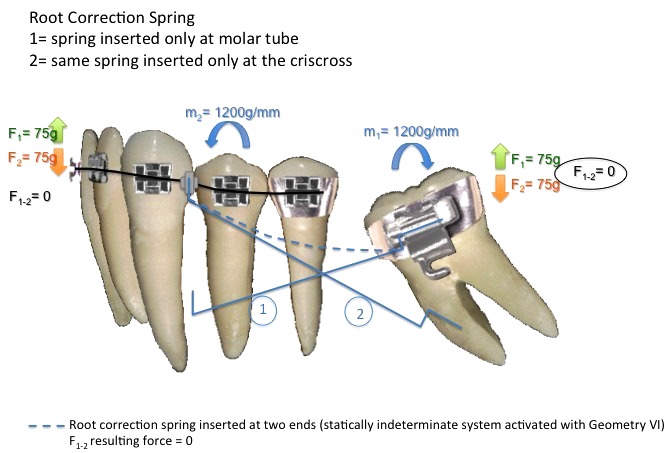
Root Correction Spring is a simplified system that works in the same way as two cantilevers, but there are two loose ends, one attached to the stainless steel wire between canine and first bicuspid, and the second end inserted at the band. Its activation promotes molar uprighting without extrusion due to the generation of a geometry VI.

Within the segmented arch technique we can use three ways of force system to bring the second molar into upright position: one cantilever - statically determinate system (Fig. **1**), two cantilevers - statically determinate system (Fig. **2**) and root correction spring - statically indeterminate system (Fig. **3**); all constructed with 0.017" X 0.025" Titanium Molybdenum Alloy or TMA (Ormco Corporation, Glendora, USA) because it is inherently more springy than stainless steel wire and provides a light continuous force distribution [7-10].

A simplified way to apply segmented arch technique in these cases would be with one cantilever, which would generate force, but most importantly, moment to tip the molar to its correct position. Along with this activation, a vertical force is generated causing the molar to erupt [7, 9], as represented in (Fig. **1**).

Second option would be double cantilever (Fig. **2**) which avoids the vertical force promoted by one cantilever system. There is one cantilever to bring the tipped molar to its right position (long one) inserted in the molar tube and attached to a point contact in the anterior part. And another cantilever (short one) that is inserted in a tube attached to anterior part and has its point contact in the tipped molar. The shorter the cantilever, the less the moment and the more the force applied. The longer the cantilever, the less the force and more the moment generated. Therefore, the short cantilever would have a greater force upon the molar, avoiding its eruption [1, 9].

**Fig. (4):**
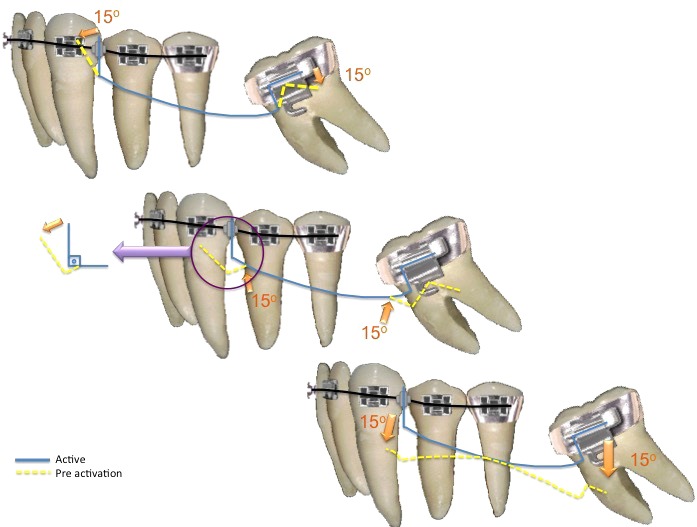
Three ways of activating the root correction spring.

A third option would be to use root correction spring, or uprighting spring, activated with geometry VI, symmetric anterior and posterior activation (Fig. **3**), that provides same force system of two cantilevers (momentum and force), generating molar uprighting and avoiding its extrusion. Symmetric anterior and posterior activations are indicated to correct inclination, no generation of vertical forces that may cause eruption of the molar or anterior segment [7] (Fig. **4**).

The purpose of this paper is to present a case successfully treated with segmented arch technique using root correction spring activated with geometry VI to promote uprighting of a tipped molar and its space closure in an adult patient.

## 2 CASE REPORT

**Fig. (5):**
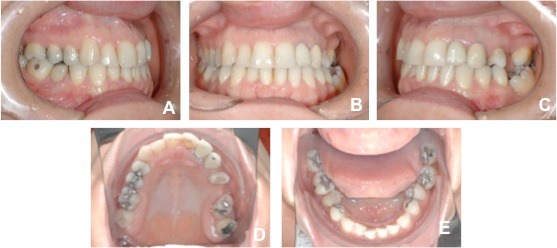
Initial intra oral photos (A.right side, B.front view, C.left side, D.upper occlusal view, E.lower occlusal view).

**Fig. (6):**
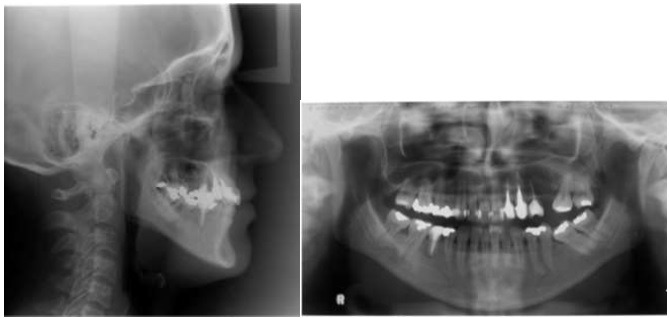
Initial panoramic and teleradiography.

An adult female patient sought orthodontic treatment in order to rehabilitate early lost teeth spaces that remained untreated. The patient presented Class I of Angle for canines and right side molars, early loss of 25, 26 and 36, light upper and lower crowding, and hiperdivergent growth pattern. The lower left second molar was found tipped over the space that once was 36, almost entirely closing the space (Figs. **5** and **6**).

**Fig. (7):**
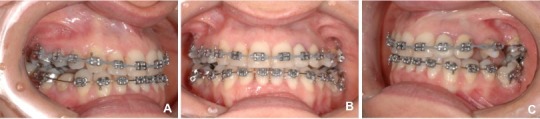
After alignment and leveling with 0.019" x 0.025" SS wires on and ready to install root correction spring (7A. right side, 7B. front view, 7C. left side where there is a segmented stainless steel wire from 46 to 35).

**Fig. (8):**
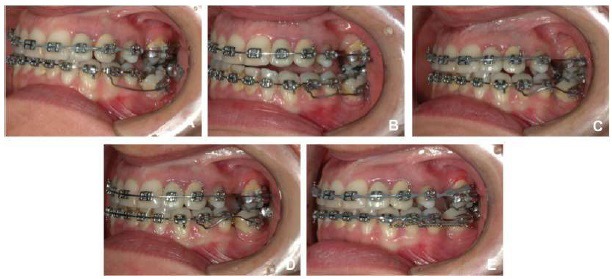
Root correction spring installed (A), after activation where Geometry VI takes place (B,C and D), and space closure with nitinol spring coil (E).

It was determined that the remaining mandibular extraction site would be closed to cut out the need for additional surgical procedures or implants, and also reduce treatment expenses. A fixed orthodontic appliance was installed (Roth prescription with .022 slot) for the alignment and leveling of the anterior and right posterior teeth, and once it was stabilized with stiff segmented wires (0.019 x 0.025-inch stainless steel), the segmented arch technique could be applied using anterior segment as anchor [10] (Figs. **7A**, **B**, **C**) . A root correction spring was constructed with 0.017" X 0.025" TMA and geometry VI was applied to it. A crisscross tube needed to be placed on the stainless steel wire between 33 and 34 in order to be an aid in holding the root correction spring that had one end clamping on to the stainless steel wire and the other end inserted into the molar tube (Fig. **8A**). An upright movement resulted from the system applied to the tipped molar; opening the space that once was first molar (Figs. **8B**, **C**, **D**). After the molar was brought to the upright position, a closed nitinol spring coil was added to close this remaining space and bring the second molar forward (Fig. **8E**).

**Fig. (9):**
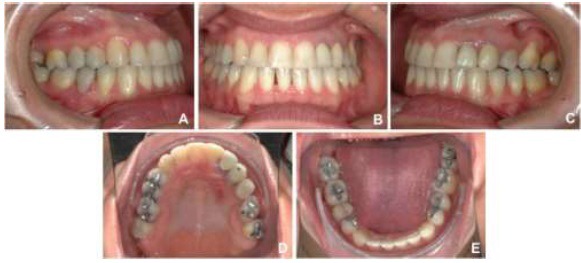
Final Intra oral photos (A. right side, B.front view, C.left side with spaces from missing teeth all closed up and in occlusion, D.occlusal upper view, E. occlusal lower view).

**Fig. (10):**
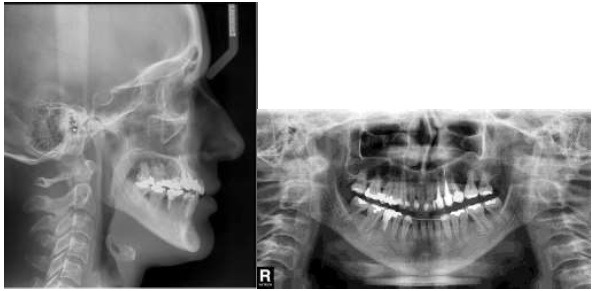
Final panoramic and tele-radiography.

This treatment ended with a Class II of Angle molar relation for the left side because the upper left second molar was also brought forward to close space left from a lost tooth. A 3x3 fixed retainer was provided for the lower arch and a removable appliance for the upper arch (Figs. **9** and **10**).

## 3 DISCUSSION

We have many treatment options today as orthodontics has evolved. Although there are many advances in material and techniques, achieving predictable and efficient tooth movement requires more than simply selecting a specific material. The fundamental basis of orthodontic therapy remains the application of mechanical forces to produce tooth movement [12]. When selecting a technique it can be determined by impaction severity, selection of opening or closing rehabilitation space, need of intrusion, as well as its simplicity and effectiveness of uprighting mechanics avoiding undesired collateral effects [9].

### 3.1 Periodontal Tissue

First thing to consider when treating an adult patient is its periodontal tissue and weather it can undergo an orthodontic treatment. Usually on tipped molar situation, there is the presence of pseudo-pockets and angular osseous crests [6,11-13] as seen in the case presented, along with occlusal collapse. For every 1mm lost in posterior contact, there is 3mm decrease in anterior segment, leading to primary occlusal trauma with potential risk of causing bone loss in anterior segment if the problem persists for a long period of time [8, 13]. Narrowing of the alveolar ridge is another thing to consider when planning on closing the edentulous space after correcting molar position; therefore, closing the extraction spaces requires remodeling of cortical bone [6].

Nevertheless, orthodontic tooth movement is an alternative method to induce bone regeneration [14]. Repositioning of tipped molars has shown to promote beneficial effects producing significant reduction in depth of existing periodontal defects and alterations to the crestal bone that accompanies tooth uprighting [4,15]. Similarly, tooth eruption and having teeth moved into adjacent osseous defects has also been reported to attain a reduction of probing depths in isolated vertical infrabony defects as well [8, 11, 15]. The case presented showed reduction of probing depth at the end.

Periodontally compromised dentitions that went through the loss of alveolar bone leads to the alteration of the center of resistance, of the involved teeth, to move apically, and the result is that teeth are more willing to tip than to move bodily [9,15,16]. For this reason, orthodontic forces in adult patients should be carefully controlled [5]; and therefore, segmented arch technique was used in this case.

### 3.2 Edentulous Space Closure

There should not be attempt to close space before full alignment of all teeth and full-sized working archwire [3], and also consider that an ideal dimension for successful mandibular first molar space closure is reported to be 6mm or less of mesiodistal space and 7mm of buccolingual width [6]. In the case presented, 4mm space was opened after molar uprighting. Bone formation is the major concern when closing an edentulous space in an adult patient. There is evidence that adults have less bone apposition when molars are moved into the narrowed space [6]. Also, this sort of movement can generate collateral effects such as the tendency for the crown to tilt mesially, apex to move in the opposite direction, and roll lingually when bound to a mesially directed force [3,16] because there is no practical way to apply a force directly through the center of resistance of a tooth to achieve pure translation without tipping. In order to avoid this, a countermoment in the form of a couple may also be applied intentionally to counteract tipping [3, 16].

On the other hand, there is also evidence that orthodontic movement is an excellent way to reclaim new alveolar bone and soft tissue when closing spaces and crestal bone damage mesially to the second molars after treatment. The bone level of the case presented was maintained after molar uprighting and space closure, which reaffirms this last statement of reclaiming new alveolar bone.

### 3.3 Force Applied

There is no agreement between studies in relation to force in this type of orthodontic movement; each author uses different forces in distinct molar uprighting techniques [9,17]. Melsen *et al.* [18] (2007) states that the optimum force has not been scientifically defined yet and that higher force levels do not necessarily gain tooth movement and that overcharging periodontal tissue can cause negative effects, such as hyalinization and ischemia therefore delaying tooth movements. The idea that the biology of each person has more influence in tooth movement than orthodontic load itself seems to be more advocated between studies [16, 18]. Among individual variations of alveolar support structures, orthodontic forces with same magnitude will cause different stress and distortion distributions in the tissues. Due to nonlinear mechanical properties of the tissues as well as the varied mechanical support of the teeth in different individuals, the load transfer through the alveolar tissues is complex and for these reasons only very small orthodontic forces should be applied to the teeth to avoid ischemia and local necrosis of the tissues [9, 18].

However, force application in cantilever mechanics is suggested to be less than 100g.mm (grams per millimeter); otherwise it can be harmful to teeth and support structures [17]. In an empirical form, the necessary moment magnitude to upright a molar varies around 1000 to 1500g.mm^9^ and one study suggests that 1200g.mm is the appropriate moment for molar uprighting [13]. As seen in Fig. (3) 1200g.mm was applied to upright the molar of the case.

### 3.4 Anchorage Unit

The strategy of uniting anterior teeth as anchor unit can prevent molar extrusion and occlusal interferences when an intrusive force on the molar is needed [2, 9]. From equilibrium, the absolute value of the opposite moment at the anterior unit must be greater than the moment at the molar. Although this might prevent molar extrusion, a larger moment at the anterior teeth can result in undesired movement of this anchorage unit [1]. Ideally, to keep the anterior unit stable during molar root correction, the center of resistance must be defined [1]. The position of the intrusive force in the anchor teeth has to be located far from the center of resistance or a moment tending to produce buccal crown movement may be produced [2, 9]. When anterior segment is united with anchorage purpose, the Center of Rotation is relocated to the molar mesial, obtaining upright effect, root mesial movement and space closure [9].

In order to avoid collateral effect on the anchor unit, the cantilever is suggested to be placed between canine and lateral incisor (Center of Resistance of anterior unit) or a little distal from Center of Resistance [9]. In the case presented, it was placed between canine and first bicuspid for patient comfort purpose.

### 3.5 Cantilevers

Cantilevers, a simple two-tooth appliance, are a statically determinant force system because the forces and the moments can be readily measured. By working with measurable forces and moments, the use of this type of appliance permits greater control by the orthodontist and improves the predictability of movement and minimizes the potential for unexpected tooth movement during orthodontic treatment [12, 16].

Its main characteristic is that there is a fixed end and it is inserted into a bracket or a tube promoting force and moment, and there is a free end that is applied to a point contact of the anterior unit that only promotes force; and there must be an equal amount of force at each end so that the moment can be produced [1, 16].

Using one cantilever system to upright a molar is effective, but a vertical force is also generated in this system causing molar extrusion [9].

An alternative to eliminate extrusion force is to use two cantilevers, where one cantilever is used as in conventional molar uprighting with one cantilever, while the other is activated to produce intrusive forces on the molar. Therefore, the extrusion effect produced by the conventional cantilever is neutralized by the second cantilever [13]. Disadvantages of this option are patient’s discomfort caused by the simultaneous use of two devices and continuous activations [1, 13].

### 3.6 Root Correction Spring

The root correction spring has more sophisticated approach for tooth movement in a single wire to deliver moments equal in magnitude and opposite in sense to both anterior and posterior units at the same time [1]. Because it is attached to both anterior and posterior units at same time, it is considered to be a statically indeterminate force system where it gets complicated to precisely determine all the forces and moments involved at both attachments at a particular time [10, 16].

Roberts *et al.* [11] (1982) described some of its advantages such as symmetrical preactivation in an extra oral procedure; easy to determine force levels; few necessary adjustments during treatment in order of load/deflection considerations in spring design; no disturbance of wire by function because of its gingival level position, and minimized patient discomfort by offsetting the spring over the edentulous ridge.

Sakima *et al.* [9] states that despite its efficiency, the root correction spring presents some disadvantages as difficulty to measure force and moment, longer treatment time and long inter bracket distance problem. However, an increase in bracket distance, as a result of segmentation and the use of auxiliary tubes enhances the points of force application and allows predictable mechanics to be executed. Moreover, increased distances allow springs to have their load deflection ratio decreased, which results in greater activations and a lower number of adjustments. Also, it allows accessories to be safely positioned and activations more safely done in comparison to conventional mechanics [13].

### 3.7 Activations

Geometries are used to understand tooth movement through appliances considered to be statically indeterminate system. For root correction spring, a symmetrical activation is needed to generate equal moments at anterior and posterior units as in Geometry VI, known as symmetrically activated. Preactivation bends such as geometries should be tested while fabricated, at insertion, and during treatment, since tooth movement alters the force system [1, 11]. In the case presented, a geometry VI was activated in order to produce symmetrical results. At the molar end of the spring, an upright and extrusion movements were produced. But since the other end of the spring was also activated with the same force, the extrusion movement was canceled. This statically indeterminate system functions as the double cantilever, a statically determinate system. The advantage of the spring is that, it is more comfortable for the patient.

### 3.8 Type of Wire Used

The more bended a wire is, for instance, a steal wire, the more force is lost for each millimeter of deactivation. On the other hand, beta-titanium wires present a lower slope of the line in deactivation, which results in a minor loss of force, *i.e.*, a lower LF ratio. Low LF ratio wires are ideal for segmented mechanics, given that they keep satisfactory levels of force during system deactivation, proving frequent follow-ups or continuous adjustments to be unnecessary [8]. Beta-titanium alloy, or TMA, have the properties of a “higher quality” wire, for instance: high springback properties, lower hardness values in comparison to steel, high formability and weldability with no reduction in resilience, and resistance to corrosion. It has higher springback properties in comparison to stainless steel and it can be deflected twice as much without permanent deformation. Additionally, it releases forces that correspond approximately to half of the forces released by steel alloys under similar activation, which causes its LF ratio to be approximately half of the stainless steel ratio [8].

### 3.9 Extrusion X Facial Pattern

The majority of uprighting appliances used produce, in addition to moment, extrusive forces. In most cases, extrusion is undesirable and results in premature contact and open bite [9]. In patients with strong muscular pattern, the occlusal force helps neutralize extrusive force. Instead, patients with fragile muscular pattern and hiperdivergent growth pattern present distress in getting intrusion component without extruding anchor teeth [9, 13]. Nevertheless, Hom and Turley [4] (1984) observed that facial pattern did not correlate with any of the other variables related to molar uprighting and space closure.

## CONCLUSION

It is shown in this case report that molar uprighting and space closure of absent molar should be taken into consideration as a potential solution. In spite of tooth movement in 3 dimensions with precise control during space closure is important in meeting treatment goals. The TMA root correction spring associated with Niti spring coil is an easy and effective orthodontic technique for closing spaces without mesial or lingual tipping, and rotation of molars by using segmented arch technique.
